# Effects of hypoxic preconditioning on memory evaluated using the T-maze behavior test

**DOI:** 10.1080/19768354.2018.1557743

**Published:** 2019-02-12

**Authors:** Yun-Hee Kim, Kuen-Su Lee, Young-Sung Kim, Yeon-Hwa Kim, Jae-Hwan Kim

**Affiliations:** a Department of Anesthesiology and Pain Medicine, Korea University Ansan Hospital, Ansan, Korea; b Department of Anesthesiology and Pain Medicine, Korea University Guro Hospital, Seoul, Korea; c Institute of Medical Science, Korea University Ansan Hospital, Korea University College of Medicine, Ansan, Korea

**Keywords:** Hypoxic preconditioning, ischemia, memory, zebrafish

## Abstract

Perioperative brain ischemia and stroke are leading causes of morbidity and mortality. Brief hypoxic preconditioning is known to have protective effects against hypoxic-ischemic insult in the brain. Current studies on the neuroprotective effects of ischemic preconditioning are based on histologic findings and biomarker changes. However, studies regarding effects on memory are rare. To precondition zebrafish to hypoxia, they were exposed to a dissolved oxygen (DO) concentration of 1.0 ± 0.5 mg/L in water for 30 s. The hypoxic zebrafish were then exposed to 1.0 ± 0.5 mg/L DO until the third stage of hypoxia, for 10 min ± 30 s. Zebrafish were assessed for memory retention after the hypoxic event. Learning and memory were tested using the T-maze, which evaluates memory based on whether or not zebrafish moves to the correct target compartment. In the hypoxic preconditioning group, infarct size was reduced compared with the hypoxic-only treated zebrafish group; memory was maintained to a degree similar to that in the hypoxia-untreated group. The hypoxic-only group showed significant memory impairments. In this study, we used a hypoxic zebrafish model and assessed the effects of ischemic preconditioning not only on histological damages but also on brain function, especially memory. This study demonstrated that a brief hypoxic event has protective effects in hypoxic brain damage and helped maintain memory in zebrafish. In addition, our findings suggest that the zebrafish model is useful in rapidly assessing the effects of ischemic preconditioning on memory.

## Introduction

The brain is one of the organs that are particularly vulnerable to ischemia. Due to their high metabolic rates, brain cells easily lose their function and die in response to hypoxia-induced ischemic insults (Murphy et al. 2008). Brain ischemia and stroke are known to be the leading causes of morbidity and mortality worldwide (Hossmann 2006; Wardlaw et al. 2010; Canazza et al. 2014). The incidence of stroke after non-cardiovascular and non-neurologic surgeries is estimated to be 0.05–7%, and the incidence after cardiac surgeries is estimated to be 2–10%. The mortality from perioperative stroke is high (Parikh and Cohen 1993; Conlon et al. 2008; Zhou et al. 2016), and, therefore, monitoring and prevention of intraoperative cerebral ischemia are very important.

Hypoxic preconditioning has a strong neuroprotective effect against cerebral ischemic injuries and perioperative cerebral ischemia/reperfusion injury (Sharp et al. 2004; Sinanović 2010). Ischemic preconditioning is a phenomenon in which short-term, non-fatal ischemia protects from later, severe, ischemic insults (Miao et al. 2010). Traditional ischemic preconditioning models include rat and rodent models that are used to investigate underlying mechanisms and neuroprotective strategies (Pan et al. 2014). Due to the importance of ischemic research, there are many basic studies investigating the degree of tissue damage in ischemic preconditioning models. However, the difficulty of assessing memory function limits research and clinical applications.

The zebrafish is a relatively small, simple organism, but because it is a vertebrate, a zebrafish gene is likely to resemble a mammalian or human gene, and similar genes may be associated with human-like function (Howe et al. 2013). Zebrafish models are being increasingly employed in neuroscience and can minimize bias in experimental results by improving ease of setting experimental conditions and observation efficiency (Shams et al. 2018). A previous study related to the zebrafish behavior change upon ischemic insult showed that hypoxia might induce a change in zebrafish brain physiology and behavior (Braga et al. 2013). These findings showed the utility of zebrafish behavior change to assess brain function, especially learning and memory. Current ischemia studies are based on histologic findings and biomarker changes, and studies regarding brain functional effects, especially memory are rare. Therefore, we evaluated whether ischemic preconditioning could attenuate brain tissue damage and preserve memory using the T-maze behavior test in zebrafish under conditions of low dissolved oxygen (DO) (Kim et al. 2017).

## Materials and methods

### Animals

This study was approved by the Ethical Committee on Animal Research at the Korea University College of Medicine (approval No. KOREA-2018-0020). In all experiments, adult zebrafish (4-6 months of age and 2.5–3.5 cm long) purchased from a local aquarium store (Jincheon, Chungcheongbuk-do, Korea) were used. The zebrafish were short-finned wild-type and had a heterogeneous genetic background. They were kept in water at 28.5°C with a light cycle of 14 h and a dark cycle of 10 h in aquarium containers and were fed brine shrimp twice a day. The aquarium container was equipped with a multistage filtration system that had a sediment filter, post-carbon filter, fluorescent UV light, and sterilizing filter (Zebrafish AutoSystem, Genomic Design, Daejeon, Korea).

### Modeling hypoxia and hypoxic preconditioning in zebrafish

**Figure 1.**
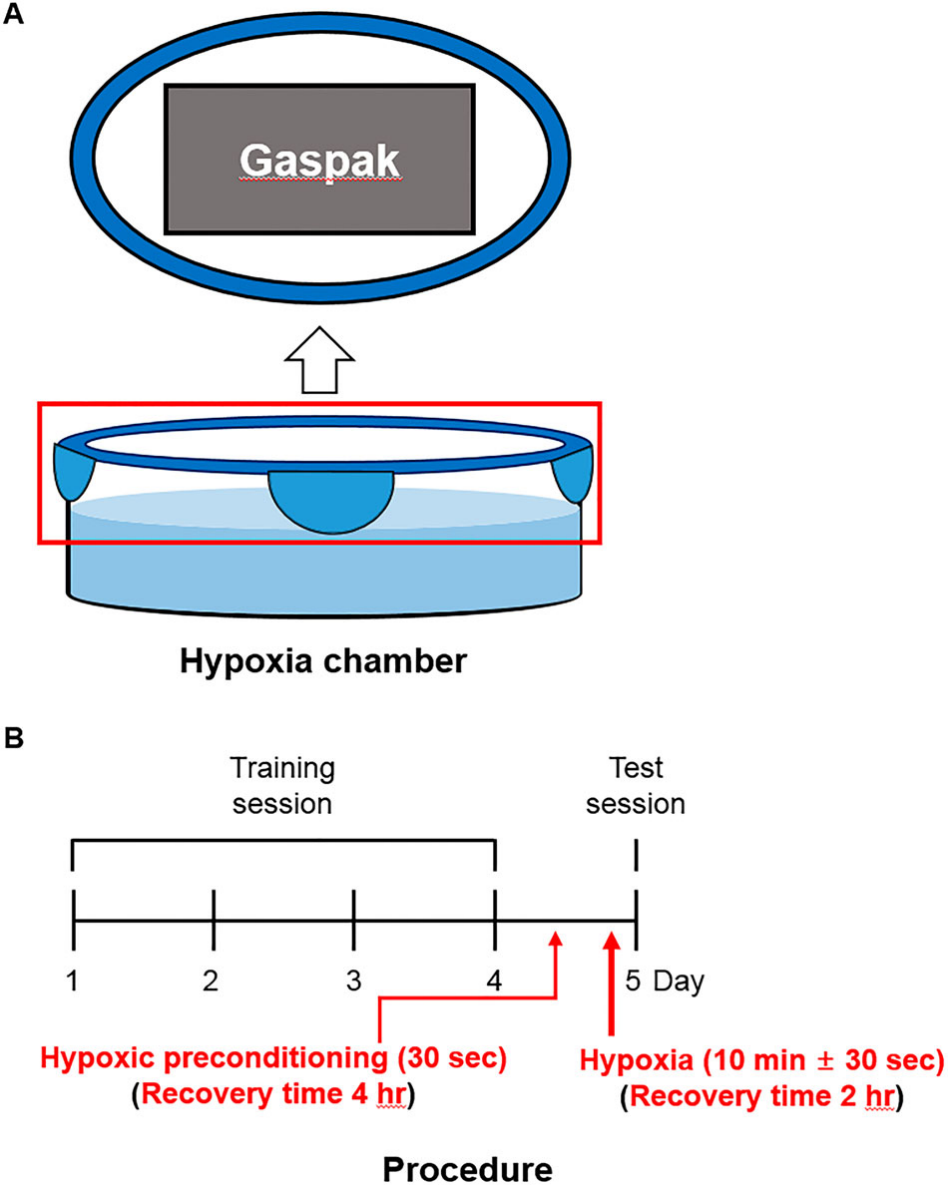
Schematic of the hypoxic chamber and procedure. (A) GasPak^TM^ attached with lid of the hypoxic chamber. The hypoxic chamber was filled with 300 mL of system water. (B) The training session was composed of 4 trials on consecutive days. The memory test was administered 24 h after the last training trial. Hypoxia was induced before the memory test and recovery was allowed for 2 h. Hypoxic preconditioning was induced before the hypoxia and recovery were allowed for 4 h.

A glass box attached to one pouch of GasPak^TM^ (BD) was used as a closed hypoxia chamber (Figure 1(A)). Hypoxia chambers were filled aquarium water that was pre-equilibrated in the hypoxia chamber for at least one night prior to zebrafish transfer to ensure appropriate hypoxic condition.

Zebrafish showed a reliable sequence of behaviors, as described previously (Braga et al. 2013). The zebrafish in the hypoxic group were exposed to hypoxic conditions up to the third stage of the hypoxia (maintenance of opercular beats with brief movements), characterized by a critical, but non-lethal, condition. The zebrafish in the hypoxic preconditioning group were exposed to hypoxic conditions for 30 s, until the first stage of hypoxia (swimming at the top). Following hypoxic incubation, zebrafish were removed from the hypoxia chamber and immediately transferred in a normoxia chamber.

### Treatment conditions and experimental groups

Animals were separated into four groups: Control, zebrafish kept in normoxic conditions for 2 h; HYPOXIA (HYP), zebrafish subjected to hypoxia followed by 2 h recovery; HYPOXIC PRECONDITIONING (HPC), zebrafish subjected to hypoxic preconditioning followed by 4 h recovery; and HPC + HYP, zebrafish subjected to a sequence of hypoxic preconditioning, 4 h recovery, hypoxia, and 2 h recovery (Figure 1(B)). After treatment, all zebrafish performed either the locomotor activity or T-maze experiments. To remove the brain, zebrafish were anesthetized using MS-222 (tricaine, Sigma-Aldrich) and euthanized by decapitation.

### TTC staining

2,3,5-triphenyltetrazolium chloride (TTC) staining was used to evaluate the activity of brain mitochondrial dehydrogenases. TTC staining was performed 2 or 4 h after the hypoxic or hypoxic preconditioning treatments. Whole brains were incubated, in darkness, with 1 mL of 2% TTC (Sigma-Aldrich, St. Louis, MO, USA) phosphate buffer saline-based solution. For staining only, brains were incubated for 40 min at 37°C. After staining, TTC solution was discarded and brains were placed in 4% paraformaldehyde overnight. Images were taken the next day. For extracting, brains were incubated for 100 min at 37°C. TTC solution was discarded after staining and brains were gently rinsed with 2–3 drops of DMSO/ethanol (1:1) solution, and then placed in 1.5 mL tubes with 1 mL DMSO/ethanol solution, in darkness, overnight. The next day, brains were removed from the tubes prior to absorbance measurements by spectrophotometer (Epoch, BioTek Instruments, USA). Brains were weighed (mg) before absorbance values were tested.

### Locomotor activity

The assessments of locomotor activities of the zebrafish included time spent mobile, meandering (absolute turn angle divided by the time mobile), and absolute turn angle (variations in the direction of the center point of the animal) in the total area of the T-maze where the zebrafish swam. Horizontal exploration represented the tendency of a zebrafish to explore whole areas. All data analyzed were from T-maze experiments. All locomotor activities were analyzed by EthoVision XT (Noldus) program.

### T-maze experiment

**Figure 2.**
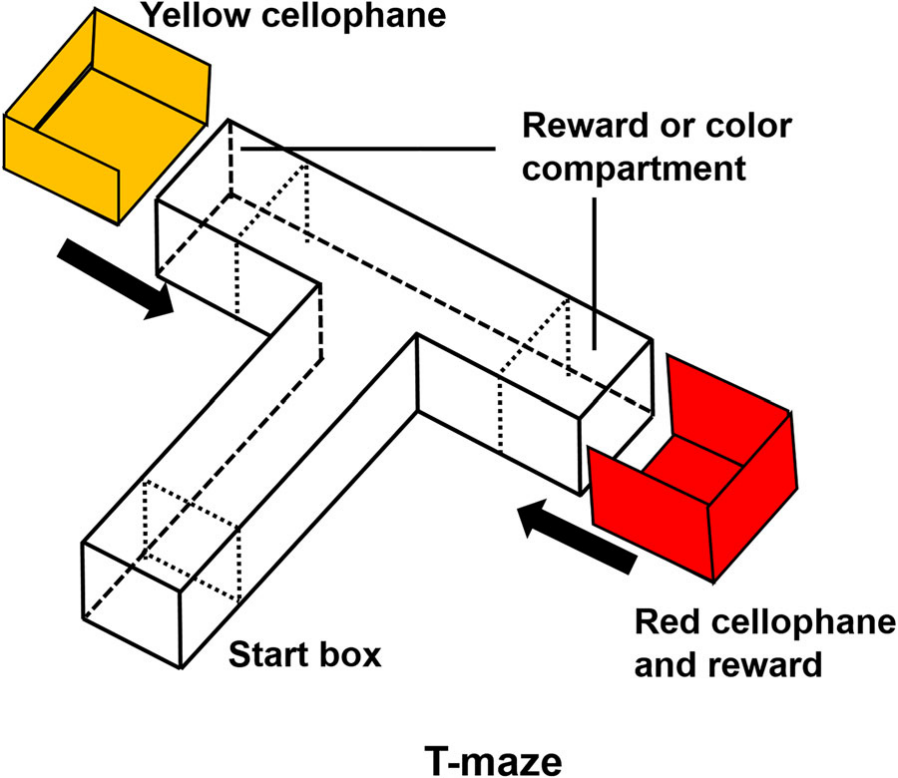
Three-dimensional T-maze. The colors indicate the two goal arms; red for the right arm and yellow for the left arm.

For learning and memory, we used the protocol as described previously, with minor modifications (Kim et al. 2017). All experiments were conducted between 10:00 and 16:00. The T-maze consisted of two arms and one stem. There was a start box (length 10 cm × width 10 cm × height 10 cm) on the bottom of the stem (50 cm × 10 cm × 10 cm) of the maze and it was divided by a transparent sliding door. Two target compartments (10 cm × 10 cm × 10 cm) were located at the end of both arms of the maze (20 cm × 10 cm × 10 cm) (Figure 2). Another transparent door was used to separate the arms of the maze from the stem. The sleeves, made of red or yellow–red cellophane, were designed to fit around the target compartments at the end of each arm.

To minimize bias, all zebrafish were subjected to a habituation trial for 2 h before testing. Each zebrafish underwent one trial per day during four consecutive training days. During training periods, 20 µL of food (brine shrimp) was placed in the red cellophane compartment before each zebrafish was placed into the start box. On the fifth day after the 4-day training period, all experimental zebrafish underwent memory testing. During the memory test, there was no colored cellophane or food reward in the T-maze. All the processes of the memory test were recorded with an OMEX camera and analyzed with an EthoVision XT (Noldus) program.

### Statistical analysis

All data were expressed as the mean (column) and standard error of the mean (error bar). The T-maze data were analyzed using the t-test or Mann–Whitney test. The locomotor activity data were analyzed using the one-way ANOVA with post hoc Bonferroni’s multiple comparison test. All data were analyzed using SPSS 20.0 software. *P* values < 0.05 were regarded as significant.

## Results

Forty-seven zebrafish were used in this study (11 control, 11 HYP, 12 HPC, and 13 HPC + HYP). The average time to reach the third stage of hypoxia in the hypoxic chamber was 10 min ± 30 s.

**Figure 3.**
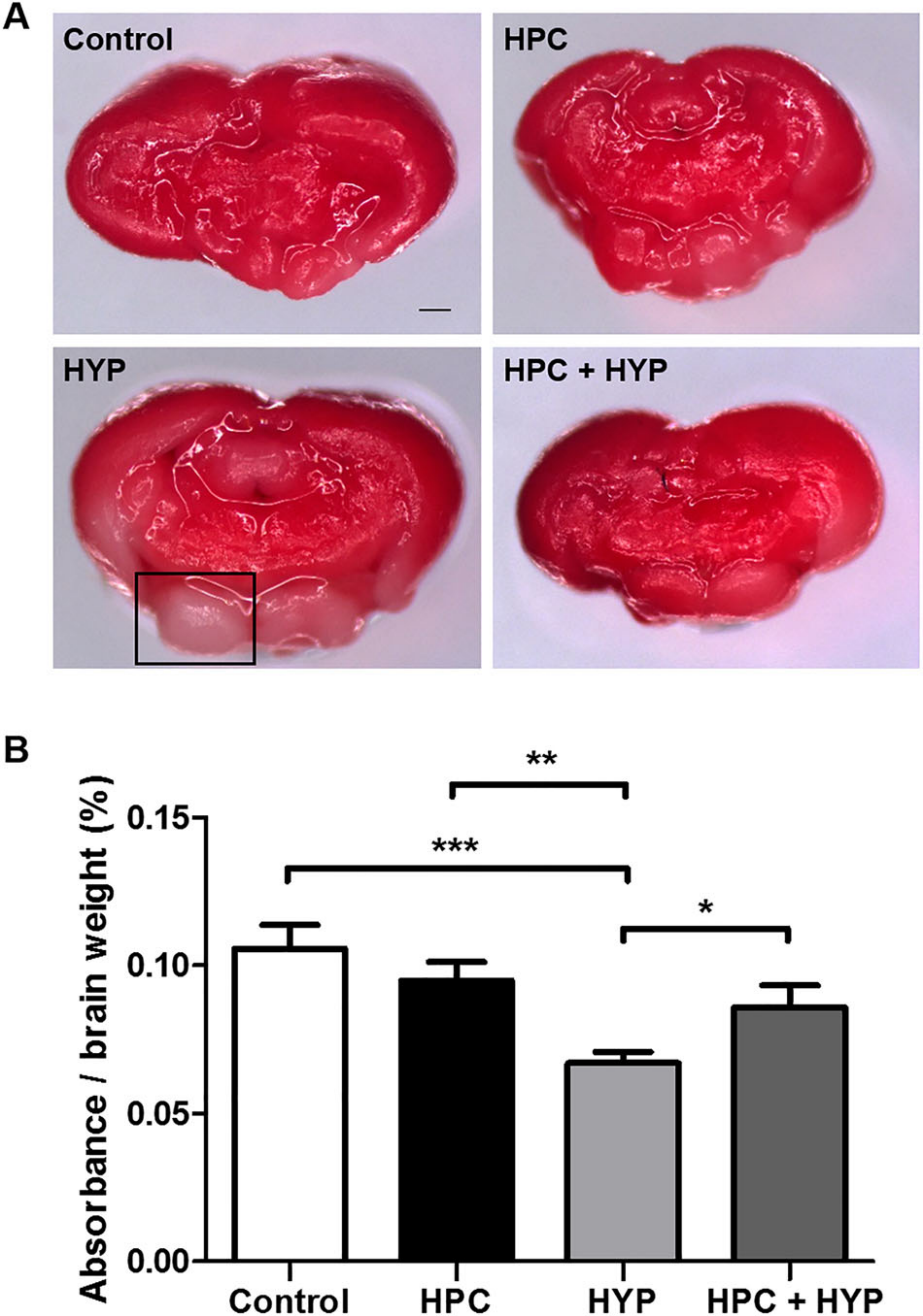
Zebrafish brain injury detected by TTC staining. (A) TTC-stained zebrafish brain sections: Control, HPC, HYP, and HPC + HYP. Scale bar, 20 μm. The square indicates an unstained area. (B) Spectrophotometric measurement (*p < .05, **p < .01, ***p < .001, each group *n* = 5). Note: TTC = 2,3,5-triphenyltetrazolium chloride, control = Zebrafish kept in normoxic conditions for 2 h, HYP = Zebrafish subjected to hypoxia and kept in normoxic conditions for 2 h, HPC = Zebrafish subjected to hypoxic preconditioning and kept in normoxic conditions for 4 h, HPC + HYP = Zebrafish first subjected to hypoxic preconditioning and kept in normoxic conditions for 4 h and then subjected to hypoxia and kept in normoxic conditions for 2 h.

TTC staining revealed deep red staining of the brains of healthy zebrafish, while hypoxic-treated zebrafish brains had more unstained areas by comparison. The ratio of absorbance to brain weight after TTC staining was significantly less in the HYP group compared with the other groups (Figure 3), while that of the HPC + HYP group was comparable to the control and HPC groups (Figure 3). These findings showed that hypoxic preconditioning treatment significantly improved TTC absorbance comparing to the hypoxic-only group.

**Figure 4.**
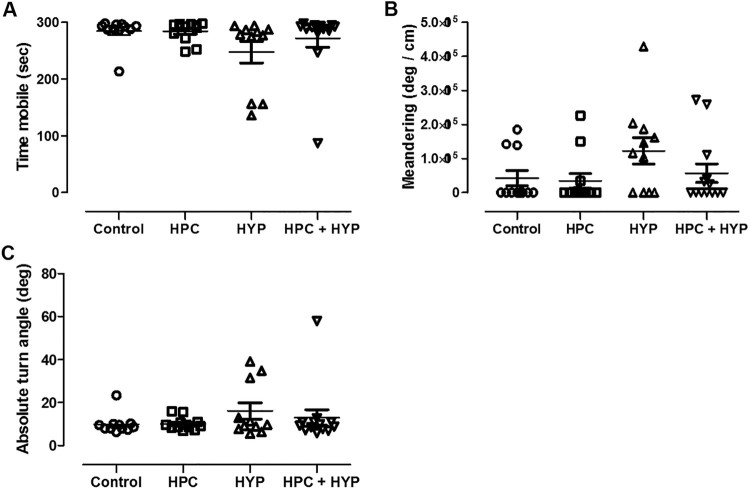
Locomotor activities including (A) the total time spent mobile, (B) absolute turn angle and (C) meandering after 2 h of recovery time. There was no difference among the groups. Note: Control = zebrafish kept in normoxic conditions for 2 h, HYP = zebrafish subjected to hypoxia and kept in normoxic conditions for 2 h, HPC = zebrafish subjected to hypoxic preconditioning and then kept in normoxic conditions for 4 h, HPC + HYP = zebrafish subjected to hypoxic preconditioning and kept in normoxic conditions for 4 h and then subjected to hypoxia and kept in normoxic conditions for 2 h.

The time spent mobile, absolute turn angle, and meandering showed no difference in all groups after 2 h of recovery time (Figure 4). These findings indicate that the results of our behavioral tests and subsequent analysis are valid.

**Figure 5.**
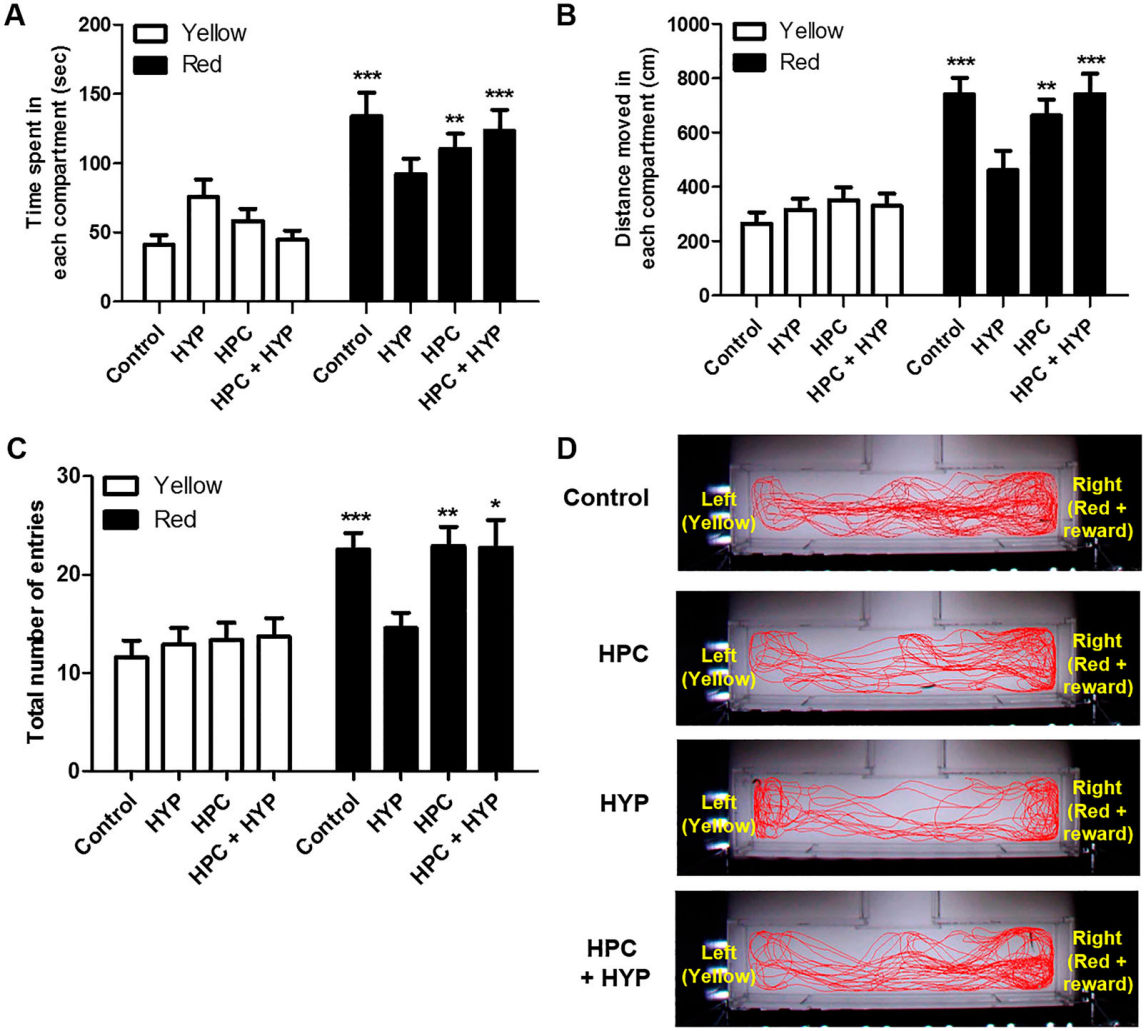
Recall ability of zebrafish in the color preference and food reward memory test. (A) Time spent, (B) distance moved and (C) the total number of entries of zebrafish in each compartment were measured in all groups. (D) The movement of each group of zebrafish and their transitions were tracked for a total of five minutes. (control, *n* = 11; HYP, *n* = 11; HPC, *n* = 12; HPC + HYP, *n* = 13). *p < .05, **p < .01, ***p < .001. Note: Control = Zebrafish kept in normoxic conditions for 2 h, HYP = Zebrafish subjected to hypoxia and kept in normoxic conditions for 2 h, HPC = Zebrafish subjected to hypoxic preconditioning and then kept in normoxic conditions for 4 h, HPC + HYP = Zebrafish subjected to hypoxic preconditioning and kept in normoxic conditions for 4 h and then subjected to hypoxia and kept in normoxic conditions for 2 h.

The control and HPC groups spent significantly more time in the red + reward compartment than in the yellow compartment compared with the HYP group (Figure 5(A), Control; ***p < .001, HPC; **p  < .01). The HYP group similarly spent time in both compartments, which indicated loss of memory. The HPC + HYP group spent significantly more time in the red + reward compartment than in the yellow compartment, as did the control group (Figure 5(A), HYP, ***p < .001). The control, HPC and HPC + HYP groups had similar results in terms of distance moved and the total number of entries as well, while the HYP group performed significantly differently in both of these measures (Figure 5(B,C)). Zebrafish in the Control, HPC, and HPC + HYP groups moved more in the compartment where red cellophane and food reward. However, hypoxic-treated zebrafish did not differentiate the distance moved in the compartment where red cellophane and food reward (Figure 5(B)) Zebrafish in the Control, HPC, and HPC + HYP groups increased the total number of entries in the compartment where red cellophane and food reward. However, the total number of entries did not differ between the right and left compartments in the HYP group (Figure 5(C)). Figure 5(D) shows the movement trends for each group. Unlike the HYP group, the control, HPC, and HPC + HYP groups showed the preference for the red and reward compartment compared to the yellow compartment, which was clearly shown in the moving trends. These findings consistently indicate that hypoxia eliminates the memory of training, and hypoxic preconditioning prevents these adverse effects and preserves memory in zebrafish.

## Discussion

Our findings suggest that hypoxic preconditioning prevents hypoxic-ischemic brain tissue and function damage, specifically, hypoxic-ischemic-induced memory deficits, as evaluated by the T-maze behavior test. Since brain hypoxia-ischemia is a common disorder with high morbidity and mortality, there have been many studies on ischemia; however, no breakthrough therapy for ischemia has yet been established. Although many researchers have made note of ischemic preconditioning, there are difficulties in confirming its protective effects in animal models. Our report is the first to confirm protective effects of ischemic preconditioning against hypoxia-induced brain damage in zebrafish in terms of memory.

In this study, we first evaluated the suitability of the zebrafish hypoxic preconditioning model. While previous studies have used a nitrogen perfusion method to set up the hypoxic chamber, we instead used GasPak^TM^. Under the hypoxic condition induced by GasPak^TM^, TTC-defined brain damage in zebrafish was observed and became worse with increasing lengths of the hypoxic period. These findings indicate a clear correlation between hypoxia duration and brain damage. In addition, zebrafish behavioral impairments, which are known to be present in hypoxic conditions, have been observed with the prolonged hypoxia in zebrafish (Braga et al. 2013). Therefore, our ischemic animal model showed results consistent with previous studies, suggesting the usefulness and effectiveness of this model for experiments designed to look at hypoxia and its effects.

A portable dissolved oxygen meter was used to confirm whether the oxygen concentration had reached 1.0 ± 0.5 mg/L DO in the hypoxia chambers. When the zebrafish reached the third stage of hypoxia (maintenance of opercular beats with brief movements), we considered it severe hypoxia and conducted the experiment (Braga et al. 2016). Cerebral injury was evaluated by TTC staining, which is a widely-used method to measure hypoxic brain damage (Yu and Li 2011). To quantify TTC staining and brain damage, the TTC stain was extracted from the zebrafish brain and the absorbance was measured using a spectrophotometric assay following standard procedures. The brains of the hypoxic preconditioning-treated zebrafish were stained less than those of the control group, but were, overall, more stained than those of the hypoxic-treated group. These results indicate that the hypoxic preconditioning treatment was protective against hypoxic-ischemic brain injury.

In this study, we focused on the utility of zebrafish as a behavioral model. Several zebrafish behavioral models have been developed and showed usefulness in evaluating cognitive function, learning, and memory (Levin and Chen 2004; Collier et al. 2014). Sison and Gerlai showed associative memory with visual perception in zebrafish (2010). Furthermore, beyond simple associative learning, spatial learning is present in zebrafish, pointing to the validity of using the zebrafish model in behavioral neuroscience. Zebrafish display rapid and reliable conditioning, well-suited for neurobehavioral tests. Moreover, T-maze method may be useful to evaluate learning, memory, and locomotor activities in zebrafish (Vignet et al. 2013; Braida et al. 2014).

Assessment of locomotor activities, including total time spent mobile, turn angle, and meandering, is useful in determining the presence or absence of abnormal movement of the zebrafish (Spink et al. 2001). Total time spent mobile represents the total time that the animal was mobile in the zone. Absolute turn angle indicates the sum of the absolute angle between each movement vector of the animal while it was inside the compartment. Meander describes the change in direction of movement of the animal relative to the distance it moves; therefore, it can be used to compare the amount of turning of animals traveling at different speeds.

A previous study, which was instrumental in our research design, showed that locomotor activities were not restored by 1 h after hypoxia, but were restored after 3 h (Braga et al. 2013). In this study, zebrafish in the hypoxic group were exposed to 10 min of hypoxia. After 2 h, the parameter values of locomotion activity, including time spent mobile, absolute turn angle, and meandering, were not different from those in the control group. In other words, the abnormal behavior of zebrafish due to hypoxic-ischemia insult was normalized to show no difference from the control group’s behaviors after 2 h of recovery time. If a zebrafish exhibits abnormal behavior, this abnormal movement itself can affect the evaluation behavior indices of zebrafish, so that the memory function measured in T-maze becomes unreliable. Therefore, we evaluated the memory of the zebrafish through behavioral assessment using T-maze after 2 h.

The principal zebrafish T-maze study is based on visual discrimination learning (Colwill et al. 2005). We used this protocol, using a T-maze apparatus with food reward and color enhancement as per our previous paper (Kim et al. 2017). In brief, the animal tested starts out at the bottom of the T (the start box on the edge of the stem). To teach it to choose the ‘correct’ target (the red compartment during the training period), positive stimuli (food reward) are used to motivate the animal. This method evaluates memory based on whether or not the zebrafish moves to the ‘correct’ target compartment. On the fifth experimental day after hypoxia and recovery periods, trained zebrafish were assessed for memory retention. In this study, we showed that memory was maintained more in the ischemic preconditioning group than in the hypoxia-only group. This confirms that ischemic preconditioning has the effect of reducing the memory impairment of zebrafish due to hypoxia.

Many clinical trials focus on remote ischemic preconditioning models because the brain is so sensitive to ischemic stimulation that it is difficult to administer proper ischemic preconditioning directly to the brain. While successful application of remote organ ischemic preconditioning has been shown in the rat model, it was hard to induce remote preconditioning in zebrafish (Dave et al. 2006). Our study focuses on the effects of ischemic preconditioning prior to global ischemia. Because zebrafish are resistant to low oxygen levels, unlike other mammals, their physiological and behavioral response physiology and may differ from other species that are unlikely to experience any hypoxic environment during adult life (Roesner et al. 2006).

Other animal models of hypoxia show brain plasticity in stroke recovery with regards to activity-dependent rewiring and synapse strengthening (Murphy and Corbett 2009). A previous zebrafish behavioral experiment (Braga et al. 2013), mentioned above, showed rapid recovery of behavioral impairment due to brain damage. Although this group suggest the occurrence of true recovery in the zebrafish hypoxic model, it is still unclear whether hypoxic preconditioning leads to resistance to hypoxic insult or promotion of damage recovery. In fact, our study considered rapid ischemic tolerance, not delayed tolerance. While rapid tolerance produces neuroprotection within 1 h of the preconditioning event, independent of new protein synthesis, classical or delayed ischemic tolerance requires protein synthesis and changes in the genomic response after 24–72 h (Caldeira et al. 2014). Further studies are required to investigate time courses and events associated with recovery in more time windows, including delayed ischemic tolerance.

To the best of our knowledge, this study is the first attempt to evaluate a behavioral model of hypoxic preconditioning in zebrafish. Zebrafish are easy to manage and our methods are easy to reproduce. Moreover, our protocol could be a useful method in future, similar experiments, or in experiments designed to confirm the protective or toxic effects of other drugs in surgery and anesthesia. Despite an abundance of research on ischemic stroke, therapeutic development is slow. Therefore, it is necessary to develop and apply more research models, and we believe our zebrafish model is a good example. Finally, this study suggests that hypoxic preconditioning can be evaluated by behavioral observation without examining brain tissue. Minimizing the damage to experimental animals, and reducing unnecessary sacrifices, may be helpful in terms of animal ethics.

In conclusion, this study suggests that a brief hypoxic state has protective effects on brain tissue, and effects on the maintenance of learning and memory, against subsequent severe hypoxic events in zebrafish. Furthermore, the zebrafish behavior model for hypoxic preconditioning is a good alternative to the conventional models.

## References

[CIT0001] Braga MM, Rico EP, Cordova SD, Pinto CB, Blaser RE, Dias RD, Rosemberg DB, Oliveira DL, Souza DO. 2013 Evaluation of spontaneous recovery of behavioral and brain injury profiles in zebrafish after hypoxia. Behav Brain Res. Sep. 15;253:145–151. doi: 10.1016/j.bbr.2013.07.019 23867150

[CIT0002] Braga MM, Silva ES, Moraes TB, Schirmbeck GH, Rico EP, Pinto CB, Rosemberg DB, Dutra-Filho CS, Dias RD, Oliveira DL, et al. 2016 Brain zinc chelation by diethyldithiocarbamate increased the behavioral and mitochondrial damages in zebrafish subjected to hypoxia. Sci Rep. 6:20279. doi: 10.1038/srep20279 26854133 PMC4745017

[CIT0003] Braida D, Ponzoni L, Martucci R, Sparatore F, Gotti C, Sala M. 2014 Role of neuronal nicotinic acetylcholine receptors (nAChRs) on learning and memory in zebrafish. Psychopharmacology. 231:1975–1985. doi: 10.1007/s00213-013-3340-1 24311357

[CIT0004] Caldeira MV, Salazar IL, Curcio M, Canzoniero LM, Duarte CB. 2014 Role of the ubiquitin-proteasome system in brain ischemia: friend or foe? Prog Neurobiol. 112:50–69. doi: 10.1016/j.pneurobio.2013.10.003 24157661

[CIT0005] Canazza A, Minati L, Boffano C, Parati E, Binks S. 2014 Experimental models of brain ischemia: a review of techniques, magnetic resonance imaging, and investigational cell-based therapies. Front Neurol. 5(19). 10.3389/fneur.2014.00019 PMC3928567 24600434

[CIT0006] Collier AD, Khan KM, Caramillo EM, Mohn RS, Echevarria DJ. 2014 Zebrafish and conditioned place preference: a translational model of drug reward. Prog Neuropsychopharmacol Biol Psychiatry. 55:16–25. doi: 10.1016/j.pnpbp.2014.05.014 24887295

[CIT0007] Colwill RM, Raymond MP, Ferreira L, Escudero H. 2005 Visual discrimination learning in zebrafish (Danio rerio). Behav Processes. 70:19–31. doi: 10.1016/j.beproc.2005.03.001 15967284

[CIT0008] Conlon N, Grocott HP, Mackensen GB. 2008 Neuroprotection during cardiac surgery. Expert Rev Cardiovasc Ther. 6:503–520. doi: 10.1586/14779072.6.4.503 18402540

[CIT0009] Dave KR, Saul I, Prado R, Busto R, Perez-Pinzon MA. 2006 Remote organ ischemic preconditioning protect brain from ischemic damage following asphyxial cardiac arrest. Neurosci Lett. 404:170–175. doi: 10.1016/j.neulet.2006.05.037 16781056

[CIT0010] Hossmann KA. 2006 Pathophysiology and therapy of experimental stroke. Cell Mol Neurobiol. 26:1057–1083. doi: 10.1007/s10571-006-9008-1 16710759 PMC11520628

[CIT0011] Howe K, Clark MD, Torroja CF, Torrance J, Berthelot C, Muffato M, Collins JE, Humphray S, McLaren K, Matthews L, et al. 2013 The zebrafish reference genome sequence and its relationship to the human genome. Nature. 496:498–503. doi: 10.1038/nature12111 23594743 PMC3703927

[CIT0012] Kim YH, Lee KS, Park AR, Min TJ. 2017 Adding preferred color to a conventional reward method improves the memory of zebrafish in the T-maze behavior model. Anim Cells Syst. 21:374–381. doi: 10.1080/19768354.2017.1383938

[CIT0013] Levin ED, Chen E. 2004 Nicotinic involvement in memory function in zebrafish. Neurotoxicol Teratol. 26:731–735. doi: 10.1016/j.ntt.2004.06.010 15451037

[CIT0014] Miao Y, Zhang W, Lin Y, Lu X, Qiu Y. 2010 Neuroprotective effects of ischemic preconditioning on global brain ischemia through up-regulation of acid-sensing ion channel 2a. Int J Mol Sci. 11:140–153. doi: 10.3390/ijms11010140 20162006 PMC2820994

[CIT0015] Murphy TH, Corbett D. 2009 Plasticity during stroke recovery: from synapse to behaviour. Nat Rev Neurosci. 10:861–872. doi: 10.1038/nrn2735 19888284

[CIT0016] Murphy TH, Li P, Betts K, Liu R. 2008 Two-photon imaging of stroke onset in vivo reveals that NMDA-receptor independent ischemic depolarization is the major cause of rapid reversible damage to dendrites and spines. J Neurosci. 28:1756–1772. doi: 10.1523/JNEUROSCI.5128-07.2008 18272696 PMC6671530

[CIT0017] Pan LN, Zhu W, Li Y, Xu XL, Guo LJ, Lu Q, Wang J. 2014 Astrocytic Toll-like receptor 3 is associated with ischemic preconditioning-induced protection against brain ischemia in rodents. PLoS One. 9:e99526. doi: 10.1371/journal.pone.0099526 24914679 PMC4051824

[CIT0018] Parikh S, Cohen JR. 1993 Perioperative stroke after general surgical procedures. N Y State J Med. 93:162–165. 8455845

[CIT0019] Roesner A, Hankeln T, Burmester T. 2006 Hypoxia induces a complex response of globin expression in zebrafish (Danio rerio). J Exp Biol. 209:2129–2137. doi: 10.1242/jeb.02243 16709914

[CIT0020] Shams S, Rihel J, Ortiz JG, Gerlai R. 2018 The zebrafish as a promising tool for modeling human brain disorders: A review based upon an IBNS Symposium. Neurosci Biobehav Rev. 85:176–190. doi: 10.1016/j.neubiorev.2017.09.002 28887224

[CIT0021] Sharp FR, Ran R, Lu A, Tang Y, Strauss KI, Glass T, Ardizzone T, Bernaudin M. 2004 Hypoxic preconditioning protects against ischemic brain injury. NeuroRx. 1:26–35. doi: 10.1602/neurorx.1.1.26 15717005 PMC534910

[CIT0022] Sinanović O. 2010 Neuropsychology of acute stroke. Psychiatr Danub. 22:278–281. 20562762

[CIT0023] Sison M, Gerlai R. 2010 Associative learning in zebrafish (Danio rerio) in the plus maze. Behav Brain Res. 207:99–104. doi: 10.1016/j.bbr.2009.09.043 19800919 PMC2814798

[CIT0024] Spink AJ, Tegelenbosch RAJ, Buma MOS, Noldus LPJJ. 2001 The EthoVision video tracking system—A tool for behavioral phenotyping of transgenic mice. Physiol Behav. 73:731–744. doi: 10.1016/S0031-9384(01)00530-3 11566207

[CIT0025] Vignet C, Begout ML, Pean S, Lyphout L, Leguay D, Cousin X. 2013 Systematic screening of behavioral responses in two zebrafish strains. Zebrafish. 10:365–375. doi: 10.1089/zeb.2013.0871 23738739

[CIT0026] Wardlaw JM, von Kummer R, Farrall AJ, Chappell FM, Hill M, Perry D. 2010 A large web-based observer reliability study of early ischaemic signs on computed tomography. The Acute cerebral CT evaluation of stroke study (ACCESS). PLoS One. 5:e15757. doi: 10.1371/journal.pone.0015757 21209901 PMC3012713

[CIT0027] Yu X, Li YV. 2011 Zebrafish as an alternative model for hypoxic-ischemic brain damage. Int J Physiol Pathophysiol Pharmacol. 3:88–96. 21760967 PMC3134003

[CIT0028] Zhou Z-B, Meng L, Gelb AW, Lee R, Huang W-Q. 2016 Cerebral ischemia during surgery: an overview. J Biomed Res. 30:83–87. 28276664 10.7555/JBR.30.20150126 PMC4820884

